# The sensitivity of TIRADS scoring on ultrasonography in the management of thyroid nodules

**DOI:** 10.12669/pjms.39.3.7313

**Published:** 2023

**Authors:** Khurshid Anwar, Adnan Yar Mohammad, Saeed Khan

**Affiliations:** 1 Khurshid Anwar, Associate Professor of ENT, ENT and Head & Neck Surgery Department, KGMC/MTI HMC, Peshawar; 2 Adnan Yar Mohammad, Associate Professor of ENT, ENT and Head & Neck Surgery Department, KGMC/MTI HMC, Peshawar; 3 Saeed Khanm, ENT and Head & Neck Surgery Department, KGMC/MTI HMC, Peshawar

**Keywords:** ACR-TIRADS, Thyroid Nodule, Ultrasonography, Biopsy, Diagnostic Accuracy

## Abstract

**Objective:**

To determine the sensitivity of ACR-TIRADS scoring by comparing its results with those obtained on biopsy of respective specimens.

**Method:**

This prospective study including N=205 patients with thyroid nodules was conducted in ENT Department of MTI Hayatabad Medical complex, Peshawar during the period from May 1, 2019 to April 30, 2022. Preoperative ultrasonography with assigning of TIRADS scores was performed in all patients. Appropriate thyroidectomies were performed in these patients and specimen biopsied. Pre-op TIRADS scores were compared with biopsy results. To determine the sensitivity of TIRADS, TR1 and TR2 were considered ‘benign’ and TR3, TR4, TR5 were considered ’malignant’ for comparison with biopsy results.

**Results:**

The mean age of patients was 37.68 years with standard deviation of ±11.52. The M: F ratio was 1:3.5. Nineteen patients (9.27%) had solitary thyroid nodules & 186 (90.73%) had multinodular goiters. TIRADS scoring was benign for 171 (83.41%) and malignant for 34 (16.58%) nodules. The biopsy results revealed 180 (87.80%) nodules to benign and the rest as malignant. The sensitivity, specificity and diagnostic accuracy were calculated at 80%, 92.77% and 91.21% respectively. Chi square test and p-value determination showed that there was a strong positive concordance between TIRADS scores & biopsy results which is highly significant (p=.001).

**Conclusion:**

The Ultrasonographic ACR-TIRADS scoring and risk stratification system is highly sensitive for detecting malignancy in thyroid nodules. It is, therefore, a reliable technique in the initial assessment of thyroid nodules and decisions can safely be based on its results. In cases of doubt, clinical judgment should be exercised before making final decision.

## INTRODUCTION

Thyroid carcinoma is one of the most common malignancies of the head and neck region. Worldwide, in 2020, the age standardized incidence rates for thyroid cancer were 10.1 per 100,000 women and 3.1 per 100,000 men and the age standardized mortality rates were 0.5 per 100,000 women and 0.3 per 100,000 men. Mortality rates were less than one per 100,000 in most countries and in both sexes. The incidence rates in women differed by more than 15 times across world regions with South Korea reaching the highest at 45 per 100,000.^1^ Early differentiated thyroid cancer is compatible with long survival, necessitating the need for a prompt diagnosis and proper management.^2^

Thyroid nodules are a common entity. Examination of autopsy specimens of those without clinical thyroid disease showed thyroid nodules larger than 1-cm in 50%. A high prevalence of palpable thyroid nodules in general population has been estimated to be 4-7%. The nodules are removed only when they are symptomatic or there is concern for malignancy.^3^,^4^ Though most goiters are benign, it is important to detect those containing malignancy. Ultrasonography is the first imaging modality used in the assessment of goiters. The presence of certain features in thyroid nodules on ultrasonography is associated with malignancy.^4^

In the past decades various thyroid imaging reporting and data systems (TIRADS) were developed for identification of malignant features in thyroid nodules. Weightage is given to ultrasonic features like composition, echogenicity, shape, margins and echogenic foci. Nodules that are solid, hypoechoic, have irregular or ill-defined margin, taller than wide, contain microcalcifications, extra-thyroidal extension are likely to be malignant. A TIRADS score is assigned to every nodule based on the presence or absence of these features. Moreover, the scores further guide the clinician as to whether a nodule should undergo an FNAC or not.^5^

Ultrasound guided FNAC helps to aspirate the suspicious nodule specifically and therefore is a useful technique that can obviate the need for unnecessary surgery on benign thyroid nodules. Currently there are various thyroid imaging reporting and data systems (TIRADS) in vogue. All represent attempts by various bodies of experts to detect malignant features in thyroid nodules on ultrasonography. In 2011, Kwak et al published a TIRADS (Kwak-TIRADS) to detect suspicious features in thyroid nodules.^6^ In 2015, the American Thyroid Association proposed a five tier risk stratification system for thyroid nodules. Simultaneously the American College of Radiology published the scoring based. ACR-TIRADS (American College of Radiology- Thyroid Imaging Reporting and Data Systems.^7^,^8^ In 2016, the Korean Thyroid Association and Korean Society of Thyroid Radiology proposed the Korean-TIRADS.^9^ Lately, in 2017, the European Thyroid Association proposed a five tier risk stratification system for thyroid nodules, the EU-TIRADS.^10^ To increase confidence of clinicians, sonographic evaluation has , now, led the researchers to compare its results with either FNA cytology or histopathology of biopsy specimens. The results are encouraging.

ACR-TIRADS scoring is widely used in our setting to categorize the thyroid nodules to assess the need for further cytological evaluation. The aim of this study was to determine the sensitivity of this risk stratification system in the initial management thyroid nodules by comparing its results directly with biopsy which is the “Gold Standard”. Comparison with biopsy result will reflect the true sensitivity of the imaging system and enhance the confidence of clinicians as only a few studies on the subject are found in our local settings. The study will add our local experience to the national and international literature.

## METHODS

This prospective and comparative study was conducted at the ENT Department of Medical Teaching Institution, Hayatabad Medical complex, Peshawar from May 1, 2019 to April 30, 2022.

The study includes 205 patients fulfilling the inclusion criteria. The sampling technique was “convenient sampling technique”. The sample size was calculated assuming 16% prevalence of thyroid nodules in general population with 95% confidence interval and 5% margin of error using Calculator.net software for sample size calculation.

### Inclusion Criteria:

- Both males and females of all ages.
- Patients with both solitary thyroid nodules and multinodular goiters.
- Thyroid nodules which shall undergo incision/or excision biopsy.

### Exclusion Criteria:

- Patients having sonograms with ACR-TIRADS scoring and FNAC only.
- Patients having sonograms with TIRADS scoring on other system(s).
- Patients with toxic solitary nodular and or multinodular goiters.
- Patients with thyroiditis.

### Data Collection Procedure:

Ethical approval for conducting the study was obtained from the institutional ethical review board through HMC-QAD-F-00 Dated 9/6/22. Patients were included in the study after taking informed consent. All the patients with goiters who reported to ENT ward, OPD and institutional based private practice were included in the study. Detailed history was obtained regarding the onset, progression and duration of thyroid gland enlargement. Enquiries were made regarding any pain, hoarseness of voice, difficulty in swallowing, breathing difficulty, intolerance to heat, palpitations, weight loss or gain, lassitude, haemoptysis and pain in the back.

A thorough clinical examination included noting the site, size, shape and number of nodules. Endoscopic direct laryngoscopy was performed to assess the mobility of vocal cords. Neck was examined for lymph node enlargement and any retrosternal extension was looked for. Signs of hypo and hyperthyroidisms were looked for. Baseline investigations were carried out in all patients to help arrive at a diagnosis and determine the patients’ fitness for subsequent thyroid surgery. In all patient’s ultrasonography of the thyroid & neck was advised with a request for making an ACR-TIRADS scoring of nodules.

Thyroid function tests including serum T_3,_ T_4_, & TSH levels were performed in all patients. ACR-TIRADS SCORING was performed by qualified radiologist holding at least a fellowship degree. The scores values given were; TR1: 0 points= benign, TR2: 2 points= not suspicious, TR3: 3 points= mildly suspicious, TR4: 4-6 points= moderately suspicious and TR5: ≥7 points= highly suspicious. Patients underwent mere observation or FNAC & observation in line with the recommendations for ACR-TIRADS score. The need for thyroid surgery was dictated by FNAC, cosmesis, patients’ symptoms and clinical suspicion of malignancy. Only the patients in whom thyroid surgery was indicated were included in the study.

All the thyroidectomy specimens were submitted for histopathology. All the biopsies were performed by qualified pathologists holding fellowship or equivalent degrees in histopathology. For the purpose of simplicity any subcategories ACR-TIRADS were omitted and the scores were categorized as the follows; TR1 and TR2 as “Benign” and TR3, TR4 & TR5 as “Malignant” for comparison with the respective biopsy result which itself was categorized either as either “Benign” or “Malignant”. The sensitivity, specificity and accuracy of ACR-TIRADS after comparison with biopsy were calculated using the formulae; Sensitivity= (TP/TP+FN) x100, Specificity= (TN/TN+FP) x100 and Accuracy= (TN+TP/TN+TP+FN+FP) x100. Where TN= True Negative, TP= True Positive, FN= False Negative and FP= False Positive.

The information obtained was recorded on a proforma. The data was analyzed using SPSS for windows. Descriptive statistics for variables like gender, age, TIRADS score and biopsy results were analyzed to determine the frequencies. Cross tables were used to find out the observed relationships of gender and ages of the patient with TIRADS score and biopsy results. The comparison of TIRADS score biopsy results was made and the accuracy of TIRADS scoring on pre-operative ultrasonography was calculated. Chi-square test was performed and p-value determined to determine the significance of the observed correlation of biopsy results and TIRADS score.

## RESULTS

The study included 205 patients. The ages ranged from 16 to 70 years with mean age of 37.68 ±11.517 years. The male to female ratio was 1:3.46. The age group wise distribution of genders is shown in Table-I. Nineteen patients had solitary thyroid nodules and 186 had multinodular goiters. The detail of TIRADS score assigned to solitary thyroid nodules and multinodular goiters are shown in Table-II. The prevalence of malignancy was higher in the solitary thyroid nodules as compared to multinodular goiters as shown in Table-III. Concordance of TIRADS scores to biopsy results has been depicted in Table-IV. The sensitivity, specificity and diagnostic accuracy were calculated at 80%, 92.77% and 91.21% respectively. Chi square test and p-value determination showed that there was a strong positive concordance between TIRADS scores & biopsy results which is highly significant (p=.001).

**Table-I:** Frequency of Gender & Age Groups.

		Gender of Patients		Total

		Male	Female	
**Age Groups**	16-25 Yrs	10	27	37(18.04%)
	26-35 Yrs	8	52	60(29.26%)
	36-45 Yrs	16	35	51(24.88%)
	46-55 Yrs	9	39	48(23.41%)
	56-65 Yrs	2	4	6(2.92%)
	66-75 Yrs	1	2	3(1.46%)

Total		46	159	205(100%)

**Table-II:** Type of Goiter & TIRADS Score Cross tabulation.

		TIRADS Score Total					Total

		TR 1	TR 2	TR 3	TR 4	TR 5	
Type of Goiter	STN	0	9	2	5	3	19
	MNG	2	160	11	5	8	186

Total		2	169	13	10	11	205

STN: Solitary thyroid nodule, MNG: Multinodular goiter.

**Table-III:** Type of Goiter & Biopsy Result Cross tabulation.

	Biopsy Result		Total

	Benign	Malignant	
Type of Goiter	STN	8	11	19
	MNG	172	14	186

Total		180	25	205

STN: Solitary thyroid nodule MNG: Multinodular goiter

**Table-IV:** TIRADS Score & Biopsy result comparison.

Frequency		Percent	p-Value
TP	20	9.8	
TN	167	81.5	
FP	13	6.3	
FN	5	2.4	

Total	205	100.0	.001

TN: True Negative, TP: True Positive, FN: False Negative and FP: False Positive.

## DISCUSSION

Nodules in thyroid gland raise concern for the presence of malignancy in the minds of both patient and clinician. As cytological evaluation used to be a norm in the assessment of thyroid nodules, the need for organized efforts at stratifying these nodules at the initial assessment was felt to obviate the need for cytological evaluation in every case. Advancement in medical imaging technology further increased the confidence of clinicians to manage thyroid nodules with more accuracy. Much work has been done in this regard in the past decade. However, until such times when enough evidence has accumulated to decide solely on the basis of image based risk stratification systems, the need for acquiring a histopathological diagnosis would be felt.^7^,^11^

Pre-operative ultrasonography of thyroid nodules can be performed by the surgeon. It will guide him to locate any suspicious nodule and aspirate it. Though the results of ultrasonography are promising but still a constant need for an adjunct histopathological evaluation is felt to determine the true nature of the disease.^12^,^13^

The results of our study show that ultrasonography is highly specific in ruling out malignancy in thyroid nodules. Out of the total 25 malignant nodules 20 (80%) were correctly identified by sonography and among the 180 benign nodules on biopsy 167 were correctly identified by sonography with a specificity of 92.77%. the false positive and false negative rates have been 6.3% and 2.4% respectively.

Whereas the number of studies comparing TIRADS scores directly with biopsy is scarce in the literature, the vast majority of studies compare sonographic scores with FNAC rather than biopsy results of resected specimens to elucidate the true nature of pathology. Al shoaibi and colleagues compared the results of ultrasonography with FNAC in management of thyroid nodules. Among 124 patients with thyroid lesions shown benign on ultrasonography, 98.38% were benign and 1.2% were malignant on FNA cytology. Among nine patients with thyroid lesions diagnosed as malignant by ultrasonography, 55.6% were confirmed to be malignant by FNA cytology and 44.4% were proved to be benign. They concluded that ultrasonography was a valuable tool and had excellent diagnostic accuracy in differentiating benign from malignant nodules.^14^

In yet another step forwards, Rahimi M and colleagues compared results of pre-operative sonography and FNA cytology with biopsy of respective specimens. Out of the 144 patients with thyroid nodules, 14 cases had suspicion of malignancy on both ultrasonography & FNAC. Thirteen of these were confirmed malignant on biopsy of resected specimens.^15^

The TIRADS scoring of thyroid nodules not only allow for risk stratification and the scoring system guides the clinician as to whether a nodule should be subjected to fine needle aspiration cytology or ‘be observed’. ACR-TIRADS is the risk stratification system widely used in our settings. Research is underway to determine the relative accuracy of various TIRAD Systems. Lately the ‘artificial intelligence’ (AI-TIRADS) based on ACR-TIRADS has been developed. Tobriner BW and colleagues found that use of artificial intelligence slightly improves the specificity while maintaining its sensitivity.^16^

Soylemiz and colleagues studied the relative accuracy of the five TIRAD Systems on 939 nodules and found the highest sensitivity (94.5%) for ACR-TIRADS. They concluded that ACR-TIRADS was the most sensitive risk stratification system for nodules of all sizes. They further stated that ACR-TIRADS has higher sensitivity than Bethesda system when compared with histopathological results.^17^

In a similar study as ours but using two TIRAD systems, Flavia Magri and colleagus conducted a prospective study on 255 patients. They compared the results of both ACR- and EU-TIRADS to results of respective biopsy specimens. The sensitivity for ACR-TIRADS and EU-TIRADS for detection of malignancy was found out to be 77.08% and 88% respectively comparable to our findings. They concluded that both of these risk stratification systems display good performance in detecting thyroid malignancy when biopsy is taken as a reference point.^18^

As image based risk stratification systems recommend if a specific thyroid nodule should be subjected to a semi invasive technique, FNAC, or be observed. Studies in the literature are abound to compare the results of these two with varying degrees of accuracy. Naushaba Malik and colleague conducted a study on accuracy of TIRADS by comparing its results with FNAC and found a significant concordance between the two. They found that thyroid nodules with TIRADS II, III and IVa showed benign cytological findings while TIRADS V had significant association with malignancy on cytology. They concluded that TIRADS classification was a reliable tool for assessment of thyroid nodules and could be used independently to determine the nature of thyroid lesion.^19^ In another similar study, Nighat and colleagues at Multan, Pakistan compared the results of TIRADS scores with FNAC. While assessing thyroid nodules in 201 patients, they found that the TIRADS sensitivity was 77.8%, specificity was 75.5%, PPV was 53.8%, and NPV 90.2%. The overall diagnostic accuracy for predicting malignancy in thyroid nodules was 76.1%. They concluded that the TIRADS score has a high diagnostic accuracy in detecting malignancy in thyroid nodules.^20^

To determine the accuracy of ACR-TIRADS, Wei li and colleagues published a meta-analysis of 16 studies in 2020 with 18614 patients and 21882 nodules which compared its scores with either FNAC, biopsy or both. The pooled sensitivity and specificity respectively were 89% and 86% with diagnostic odds ratio of 18.46. They concluded that the use of ACR-TIRADS could avoid a large number of unnecessary biopsies although, at the cost of slight decline in sensitivity.^21^ Zeeshan Jamal and colleagues conducted a similar multicenter study comparing the results of FNAC, sonography and biopsy. They concluded that sensitivity of FNAC and ultrasonography was equal; however, FNAC was more specific than sonography.^22^

The present study is based on objective assessment of thyroid nodules in our settings. The results obtained on ultrasonography have directly been compared with biopsy which is the gold standard. It will help the clinicians to make safe decisions with more confidence. We advocate that histopathological examination of all types of thyroid nodules is unnecessary albeit suggested by TIRADS scores. This will save time and money while not compromising the safety of patients at the same time. It will also curtail the number of unnecessary surgeries and reduce burden on the laboratory services. Moreover, it will contribute our experience to the literature on this subject.

### Limitations:

Besides the sample size being small, ultrasonography of thyroid nodules is highly operator dependent. This study compares the results of TIRADS scores with biopsy results whereas most studies compare them with FNAC so a direct comparison is difficult to be drawn. Further multicenter studies and meta-analyses are required to determine the efficacy of ultrasonography using TIRADS scores in the assessment of thyroid nodules in our settings.

## CONCLUSION

The Ultrasonographic ACR-TIRADS scoring and risk stratification system is highly sensitive for detecting malignancy in thyroid nodules. It is, therefore, a reliable technique in the initial assessment of thyroid nodules and decisions can safely be based on its results. We recommend that all thyroid nodules should be assessed using this risk stratification system but in cases of doubt, clinical judgment should be exercised before making final decision.

### Authors’ contribution:

**KA:** Conception of idea, acquisition of data, analysis & interpretation of data, drafting the article, critical revision and final approval for submission and is accountable for integrity of data.

**AYM:** Acquisition of data, analysis of data, drafting the article and helped in data analysis.

**SK:** Acquisition of data, analysis of content and formulations of tables, drafting the article.
